# Supplementary material for: High‐throughput CRISPRi phenotyping identifies new essential genes in Streptococcus pneumoniae
Source: Mol Syst Biol. 2017 May 10;13(5):931. doi: 10.15252/msb.20167449 (PMC5448163; doi:10.15252/msb.20167449)
Supplement: Supplementary file 1 — Appendix [file MSB-13-931-s001.pdf]

Appendix for:

High-throughput CRISPRi phenotyping identifies new essential genes in *Streptococcus pneumoniae*

Xue Liu[1,2], Clement Gallay[1], Morten Kjos[1,3], Arnau Domenech[1], Jelle Slager[1], Sebastiaan P. van Kessel[1], Kèvin Knoops[4], Robin A. Sorg[1], Jing-Ren Zhang[2] and Jan-Willem Veening[1,5]*

[1]Molecular Genetics Group, Groningen Biomolecular Sciences and Biotechnology Institute, Centre for Synthetic Biology, University of Groningen, Groningen, The Netherlands
[2]Center for Infectious Disease Research, School of Medicine, Tsinghua University, Beijing, China
[3]Department of Chemistry, Biotechnology and Food Science, Norwegian University of Life Sciences, N-1432 Ås, Norway
[4]Molecular Cell Biology, Groningen Biomolecular Sciences and Biotechnology Institute, University of Groningen, Groningen, The Netherlands
[5]Department of Fundamental Microbiology, Faculty of Biology and Medicine, University of Lausanne, Biophore Building, CH-1015 Lausanne, Switzerland

Correspondence to Jan-Willem.Veening@unil.ch

Table of Contents

Appendix Methods **3**
Strain construction 3
RNA sequencing 4
Data analysis on growth of the CRISPRi library 5
Identification of suppression mutation of CRISPRi system 6
Quantify *oriC-ter* ratio by qPCR 7
In-gel GFP fluorescence scanning 7
Appendix Figures **9**
Figure S1. Polar effects of the CRISPRi system 9
Figure S2. Microscopy of CRISPRi strains with sgRNA targeting genes involved in chromosome biology. 11

Figure S3. Microscopy of CRISPRi strains with sgRNA targeting genes involved in transcription. 12
Figure S4. Microscopy of CRISPRi strains with sgRNA targeting genes involved in translation. 12
Figure S5. Microscopy of CRISPRi strains with sgRNA targeting genes involved in cell membrane biosynthesis. 13
Figure S6. Microscopy of CRISPRi strains with sgRNA targeting genes involved in cell division 14
Figure S7. Microscopy of CRISPRi strains with sgRNA targeting genes involved in capsule synthesis. 15
Figure S8. Microscopy of CRISPRi strains with sgRNA targeting genes involved in peptidoglycan synthesis. 16
Figure S9. Microscopy of CRISPRi strains with sgRNA targeting genes involved in teichoic acid biosynthesis. 17
Figure S10. Growth profiles and morphological changes of CRISPRi strains with sgRNA targeting the hypothetical genes studied in this work. 18
Figure S11. Functional verification of *pcsB*, *vicR, divIC and rafX* by CRISPRi. 19
Figure S12. Annotation and characterization of *dnaD*, *dnaB*, *yabA* as important genes of chromosome biology. 20
Figure S13. In-gel fluorescence scanning of TarQ-GFP, TarP-GFP, GFP-MurT and GFP-GatD. 21
Figure S14. Western blotting to detect phosphorylcholine-containing molecules of S. pneumoniae. 21
Appendix Table 23
Appendix References 25

Appendix Methods

Strain construction

luc reporter strain. The *luc* gene was amplified from pPEP23 (Sorg et al, 2015) with primers MCS_R and luc_R2_BamHI, and subcloned into pPEPY (Veening laboratory collection) by EcoRI/BamHI digestion and ligation, and then transformed into strain DCI23 to construct strain XL30. After that, XL30 was transformed with plasmid pPEPX or pPEPX-P3-sgRNA*luc*, making strains XL28 and XL29, respectively.

GFP fusion strains. To make C-terminal GFP fusions, the genes were amplified with the primers listed in Table EV4, and then the amplicons were digested with NotI and SpeI, ligated into pMK17 (Veening laboratory collection), and transformed into *S. pneumoniae* D39. For N-terminal GFP fusions, the amplicons were cloned into pCG6 (Veening laboratory collection). All strains are listed in Table EV5.

Gene deletions. To delete *spd_1197*, *spd_1198*, *spd_1416* and *spd_1417*, up- and down-stream sequences of the genes were amplified with the primers described in Table EV4. An erythromycin resistance marker was amplified with primer pair Ery_F_gibson/Ery_R_gibson from pJWV502 (Beilharz et al, 2015). Next, the amplified upstream sequence, erythromycin resistance marker and downstream sequence were fused by Gibson assembly using DNA assembly master mix (NEBuilder HiFi DNA Assembly Master Mix, Cat. E2621S). To obtain the depletion strains, the assembly products were transformed into strains containing the Zn^{2+}-inducible GFP-fusions of the respective genes using 0.1 mM Zn^{2+} in the growth medium.

To make depletion strains of *spd_1405* and *spd_1522*, an extra copy of the gene controlled by a Zn^{2+}-inducible promoter P_{Zn} was inserted into the *bgaA* locus of

S. pneumoniae D39. First, *spd_1405* and *spd_1522* were amplified with primers spd_1405_For/spd_1405_Rev, spd_1522_For/spd_1522_Rev, respectively. Then the amplicons were digested with EcoRI and SpeI, and ligated into pMK11 (Kjos et al, 2016), and transformed into *S. pneumoniae* D39, leading to construction of strains XL35 and XL38. The native *spd_1405* and *spd_*1522 genes were replaced by an erythromycin resistance marker as described above. To make the knockout mutants of *yabA*, *metA*, *hpt*, *spd_1895*, *eep*, *spd_1520* and *spd_0379*, up- and down-stream sequences of the target genes were amplified with primer pairs as described in Table EV4. An erythromycin resistance marker was amplified with ery_start/ery_end (for *yabA*) or Ery_F_gibson/Ery_R_gibson (for the others) from pJWV502 (Beilharz et al, 2015), and fused with the up- and downstream amplicons by Gibson assembly. The Gibson assembly products were subsequently transformed into *S. pneumoniae* D39 and the transformants were selected with erythromycin.

RNA sequencing

S. pneumoniae strains XL28 and XL29 were grown to OD_{600}=0.4 in 5-ml tubes at 37ºC, and then diluted 1:100 in 20 ml fresh C+Y medium with (both XL28 and XL29) or without (only XL28) 1 mM IPTG (two replicates per condition). The cultures were incubated at 37ºC for 2.5 hours before growth was stopped on ice and cells collected by centrifugation at 4°C, 8000 rcf, 10 min. The pellets were washed with cold TE buffer (10 mM Tris-Cl, pH 7.5. 1 mM EDTA), quickly frozen in liquid nitrogen and then stored at -80ºC. Cells were lysed by bead beating in TE buffer and RNA was isolated as described previously (Slager et al, 2014). The purity and integrity of all isolated RNA samples were assessed by RNA gel electrophoresis (Aranda et al, 2012). Ribosomal RNA was removed from total RNA with the RiboZero rRNA Removal Kit (Illumina, US). The processed RNA samples were used to prepare double-stranded

complementary DNA (cDNA) libraries with the TruSeq® Stranded Total RNA Sample Preparation Kit (Illumina, US) according to the manufacturer's protocol and sequenced on an Illumina HiSeq2000 in 50 nt SE mode. The quality of raw reads was checked by FastQC v0.11.5 (Babraham Bioinformatics, UK), based on which read trimming was performed. Adapters were removed and, with Rockhopper 2.0.3, the trimmed reads were mapped to the reference genome of *S. pneumoniae* D39 (accession NC_008533, assembly GCF_000014365.1, date of download 9th Nov. 2016) modified with all the insertions present (*lacI*, *sgRNA*, *dcas9*) in strains XL28 and XL29. Differential expression analysis was performed with the T-Rex pipeline (de Jong et al, 2015) and the result was plotted as a volcano plot. The significantly changed genes are listed in Table EV2.

Data analysis on growth of the CRISPRi library

The original CRISPRi library was constructed by picking 3 colonies from the transformation plates for each of the target genes, resulting in a library size of 1173 strains. As described above, growth assays for each of these strains were performed in the presence and absence of IPTG. Analysis of the obtained data was performed using RStudio (RStudio, 2015). Firstly, for each condition (strain + IPTG concentration), if applicable, a representative growth curve was selected from three replicates by first eliminating clear outliers by visual inspection and subsequently randomly selecting one of the remaining replicates. Secondly, each of the 1173 datasets were analyzed for two types of 'defects': either a OD-difference of more than 4-fold between induced and uninduced cells, or an increased-lysis phenotype. For qualification as increased-lysis we accepted either a drop of OD_{595} below 70% of the maximum of that specific condition, or a negative slope during the last 90 minutes of measurement that exceeds a specific threshold (-0.05 log_2 OD points/hour). The

latter was assayed by creating a least square fit to the last 10 data-points. We then had to account for the fact that each colony picked in the library preparation had a certain probability to be the result of failed cloning. We used this fact to select a group of 36 negative control strains, by identifying strains that did not show any phenotype, while the other 2 strains obtained for the knockdown of the same gene displayed a clear OD-difference phenotype. Furthermore, we used the described phenotype analysis to select one representative dataset (growth with or without IPTG) for each of the 391 genes under study, according to the following hierarchy: I) if one or more out of the three strains corresponding to the gene showed an OD-difference phenotype, these strains were assumed to be true transformants and a random dataset from these was selected as representative. II) if none of the three strains displayed an OD-difference phenotype, but one or more showed increased lysis, these were assumed to be true transformants and a representative dataset was again selected randomly. III) if none of the three strains showed a OD-difference or increased lysis, a random dataset was selected from the three datasets. Based on this, a single transformant of each of the 391 sgRNA was sequenced. The sequencing result showed that 348 of the 391 sgRNAs (89%) were confirmed as functional sgRNAs. The additional 43 strains are in the process of being reconstructed and re-sequenced to complete the CRISPRi library for future research.

Identification of suppression mutation of CRISPRi system

T2 cells of the CRISPRi strains were inoculated into fresh C+Y medium with 1 mM IPTG by 1:1000. Before streaked onto agar plate, the bacterial culture was incubated at 37ºC for 15 hours. Single colonies were picked and regrew in fresh C+Y. To identify mutations, *dcas9* and *sgRNA* were amplified with primer pairs

Ppepx1_F/bga_down_R and PpepX1_F/PpepX1_R from genome of the suppressor mutants, respectively, and analyzed by sequencing.

Quantify *oriC-ter* ratio by qPCR

T2 cells of wild-type *S. pneumoniae* D39 and related mutants were thawed and diluted with fresh C+Y by 1:100 (0.1 mM $ZnCl_2$ was supplied for depletion strains) and incubated at 37ºC. When OD_{600} reached 0.1, cells were spun down, washed with fresh C+Y, and resuspended with an equal volume of fresh C+Y medium. The resuspended bacterial culture was diluted 1:10 into fresh C+Y with or without 0.1 mM $ZnCl_2$, followed by incubation at 37ºC for 2.5 hours. After that, cells were collected and washed with TE buffer and stored at -80°C. Isolation of genomic DNA of *S. pneumoniae* was performed with the Wizard Genomic DNA Purification Kit (Promega). For cell lysis, 0.05% SDS, 0.025% sodium deoxycholate and 200 µg/ml RNase were added into the nuclei lysis buffer of the kit. DNA was dissolved in deionized water. Primer pair “parB prote forward/parB prote reverse” amplifying a sequence near to *oriC*, and “ter Sp forward/ter Sp reverse” amplifying a sequence near to *ter*, were used to determine the *oriC-ter* ratio by qPCR. 8.8 µg of DNA, 0.6 pmol of each primer and 10 µl of SYBR Green Supermix (Bio-Rad) were added into the 20 µl of qPCR reactions. Amplification was performed on an iQ5 Real-Time PCR detection system (Bio-rad) with the following program: 95ºC for 3 min, 40 cycles of 95ºC for 30s, 60ºC for 30s, 72ºC for 30s. Analyses of the data were performed as described previously (Slager et al, 2014).

In-gel GFP fluorescence scanning

T2 cells of *S. pneumoniae* strains were inoculated into C+Y medium with 0.1 mM Zn^{2+} by 1:50 dilution, and then incubated at 37ºC. When OD_{600} reached 0.3, 3 ml of

bacterial culture was centrifuged at 8000 rcf for 2 min, and then washed with 1 ml of cold TE buffer (10 mM Tris-HCl, pH 7,5; 1 mM EDTA, pH 8.0). The pellets were resuspended in 150 μl of TE buffer and sonicated until the solution became clear. The sonicated cell lysates were centrifuged at 12000 rcf for 5 min. Then 100 μl of supernatant was mixed with 100 μl of 2×SDS loading buffer (100 mM Tris-HCl, pH 6.8; 4% SDS; 0.2% bromophenol blue; 20% glycerol; 10 mM DTT), and incubated at room temperature for 5 min, after which, 15 μl of sample was loaded into each well of 12% SDS-PAGE for protein separation. GFP fluorescence signal was captured by Typhoon 9400 variable mode imager (GE).

Appendix Figures

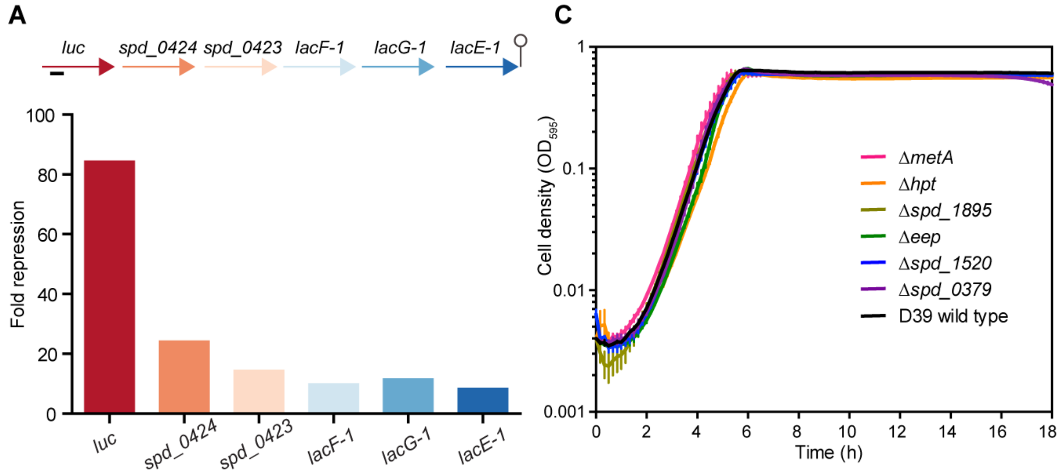

B

Gene name	Transformants (1 µl)	Transformants (10 µl)	Transformants (100 µl)
metA	100	>100	>1000
hpt	72	>100	>1000
spd_1895	3	>100	>1000
eep	60	>100	>1000
spd_1520	34	>100	>1000
spd_0379	14	>100	>1000
spd_1198 (essential gene)	0	5	10

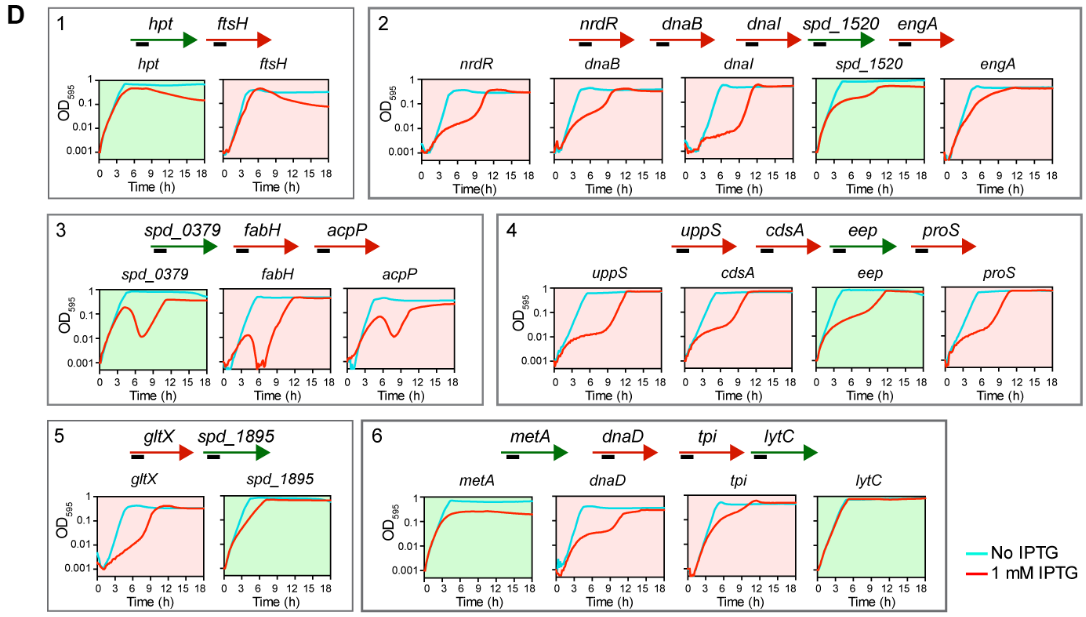

Figure S1. Polar effects of the CRISPRi system

A. CRISPRi repression of *luc* and its downstream genes detected by RNA-Seq analysis. Related to Figure 1D. Gene *luc* is co-transcribed with the downstream genes (*spd_0424*, *spd_0423*, *lacF-1*, *lacG-1* and *lacE-1*) with a terminator after *lacE-1*. The short black line

represents the binding site of the sgRNA. Y-axis represents the repression fold analyzed by RNA-Seq (data in Table EV2).

B-C. Genes *metA*, *hpt*, *spd_1895*, *eep*, *spd_1520*, and *spd_0379* are dispensable in *S. pneumoniae* D39. (**B**). Table shows the number of transformants obtained from gene deletion transformation plating with different volume of culture. A known essential gene *spd_1198* was included as reference. (**C**). Growth analysis of the deletion mutants compared with wild-type *S. pneumoniae* D39. The averages of triplicates with SEM were used for plotting.

D. Growth phenotypes of CRISPRi strains targeting genes in the operons consisting of both essential and dispensable genes. Six operons were selected (number 1-6). The green background represents growth curve of CRISPRi strains targeting dispensable genes; red background represents growth curve of CRISPRi strains targeting essential genes. The black short lines represent the position of the sgRNA binding site.

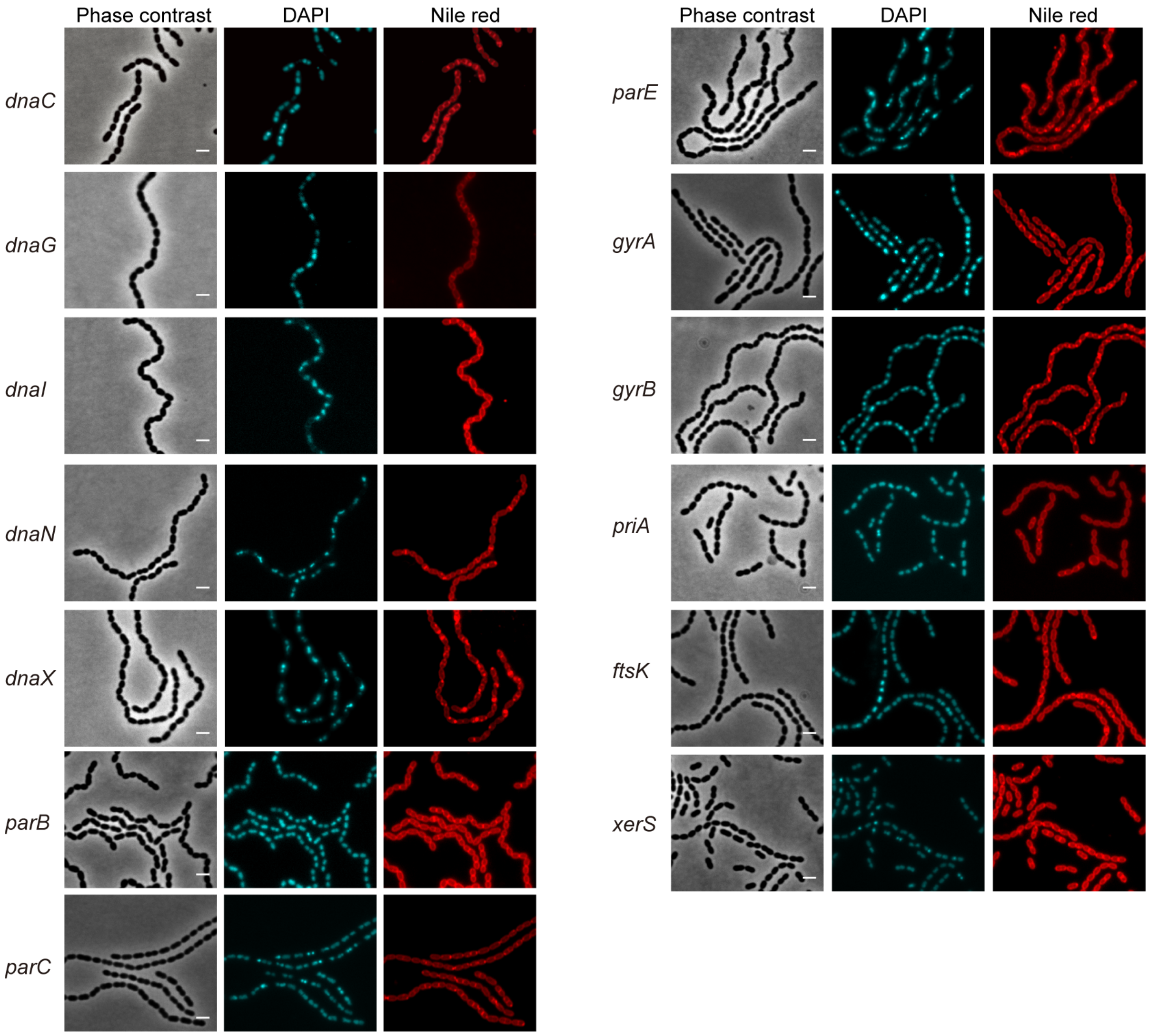

Figure S2. Microscopy of CRISPRi strains with sgRNA targeting genes involved in chromosome biology. Representative images of phase contrast, DAPI staining and nile red staining are shown. Scale bar = 2 μm.

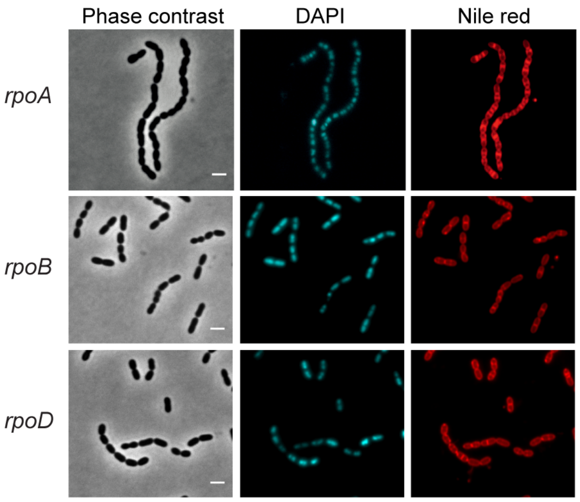

Figure S3. Microscopy of CRISPRi strains with sgRNA targeting genes involved in transcription. Representative images of phase contrast, DAPI staining and nile red staining are shown. Scale bar = 2 μm.

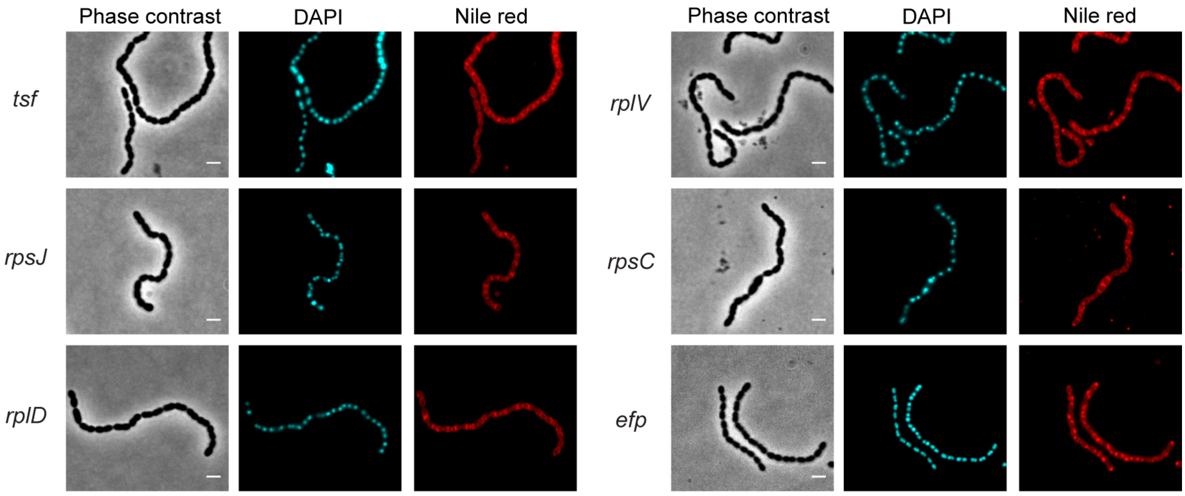

Figure S4. Microscopy of CRISPRi strains with sgRNA targeting genes involved in translation. Representative images of phase contrast, DAPI staining and nile red staining are shown. Scale bar = 2 μm.

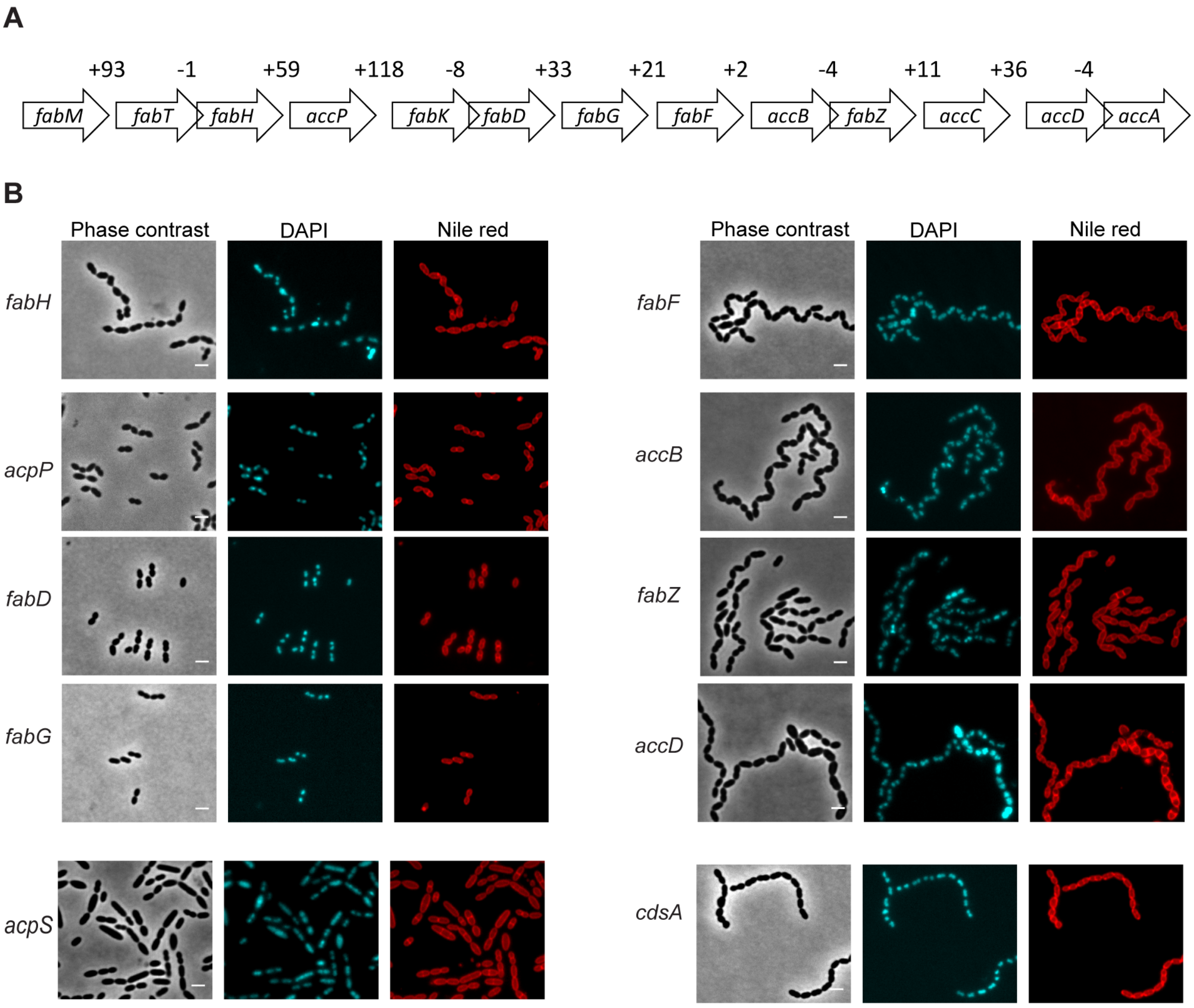

Figure S5. Microscopy of CRISPRi strains with sgRNA targeting genes involved in cell membrane biosynthesis.

A. Gene cluster of fatty acid biosynthesis of *S. pneumoniae* D39, "+" denotes the interspace between neighboring genes, and "-" denotes the overlap between neighboring genes in base pairs.

B. Representative images of phase contrast, DAPI staining and nile red staining are shown. Scale bar = 2 μm.

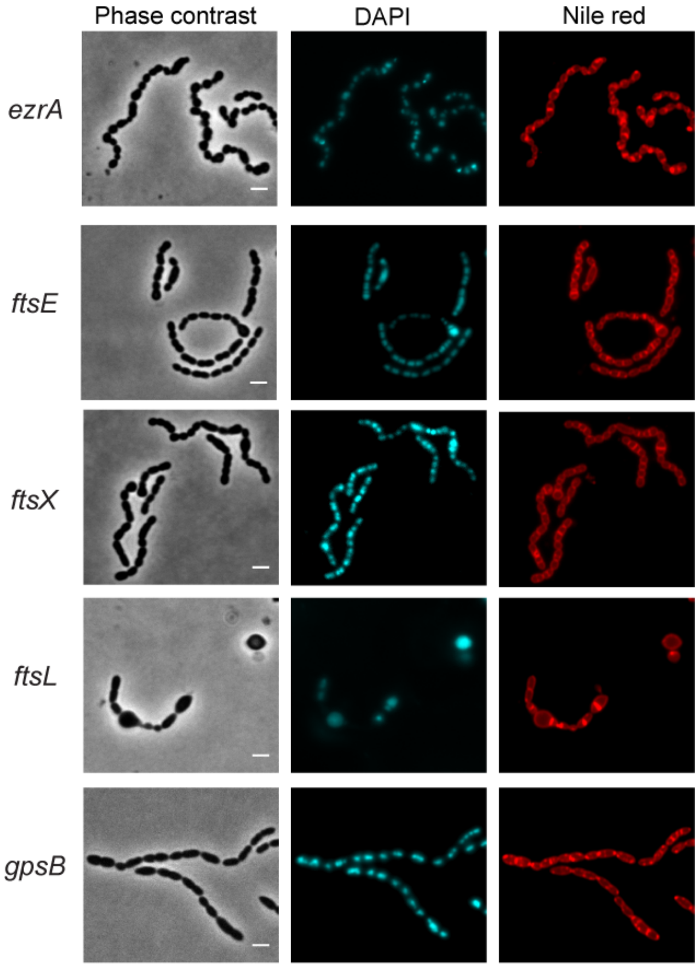

Figure S6. Microscopy of CRISPRi strains with sgRNA targeting genes involved in cell division. Representative images of phase contrast, DAPI staining and nile red staining are shown. Scale bar = 2 µm.

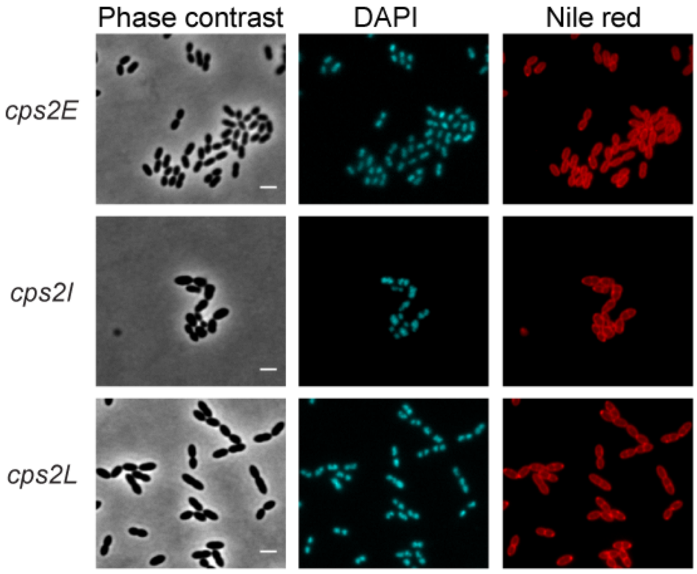

Figure S7. Microscopy of CRISPRi strains with sgRNA targeting genes involved in capsule synthesis. Representative images of phase contrast, DAPI staining and nile red staining are shown. Scale bar = 2 μm.

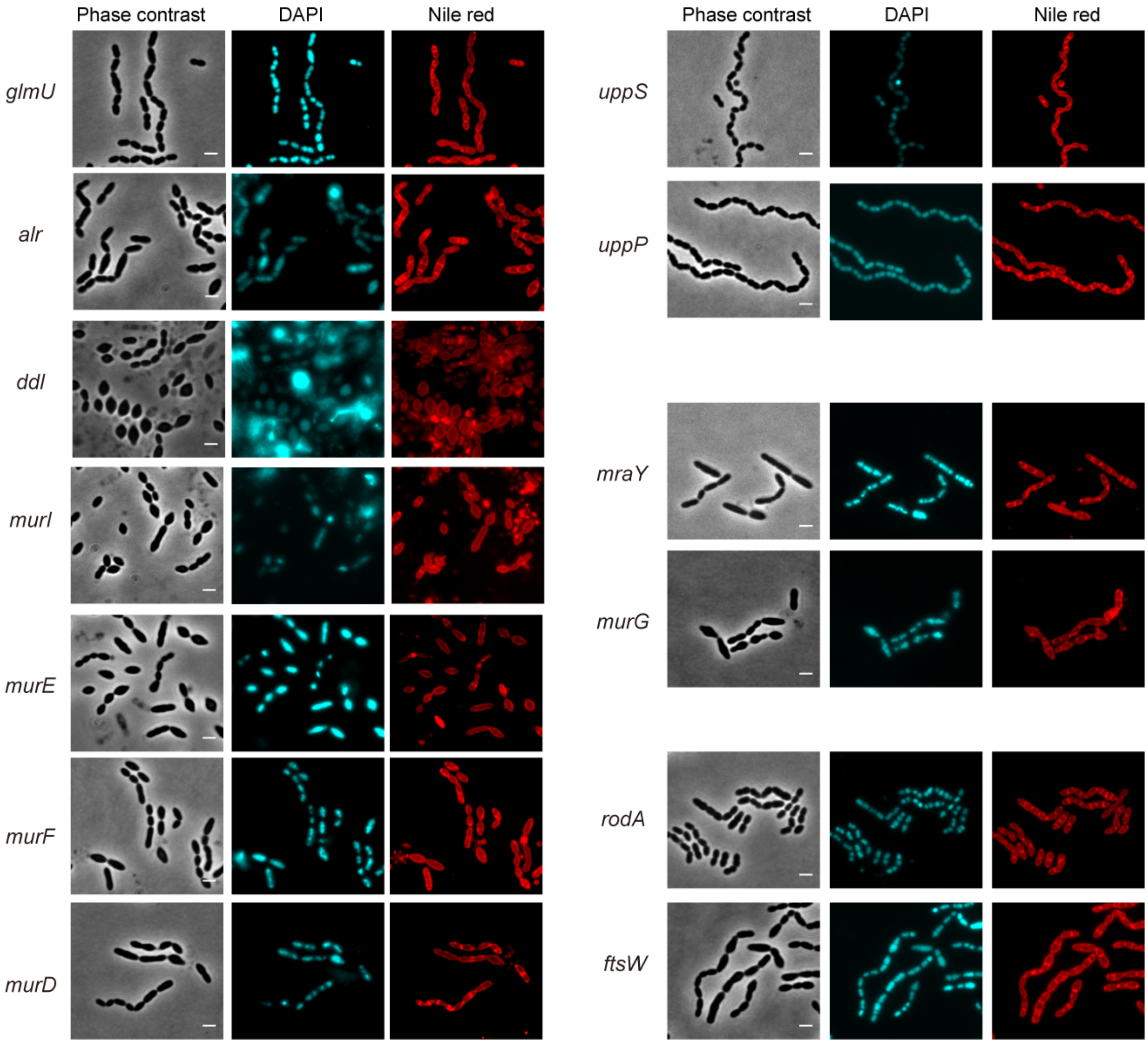

Figure S8. Microscopy of CRISPRi strains with sgRNA targeting genes involved in peptidoglycan synthesis. Representative images of phase contrast, DAPI staining and nile red staining are shown. Scale bar = 2 μm.

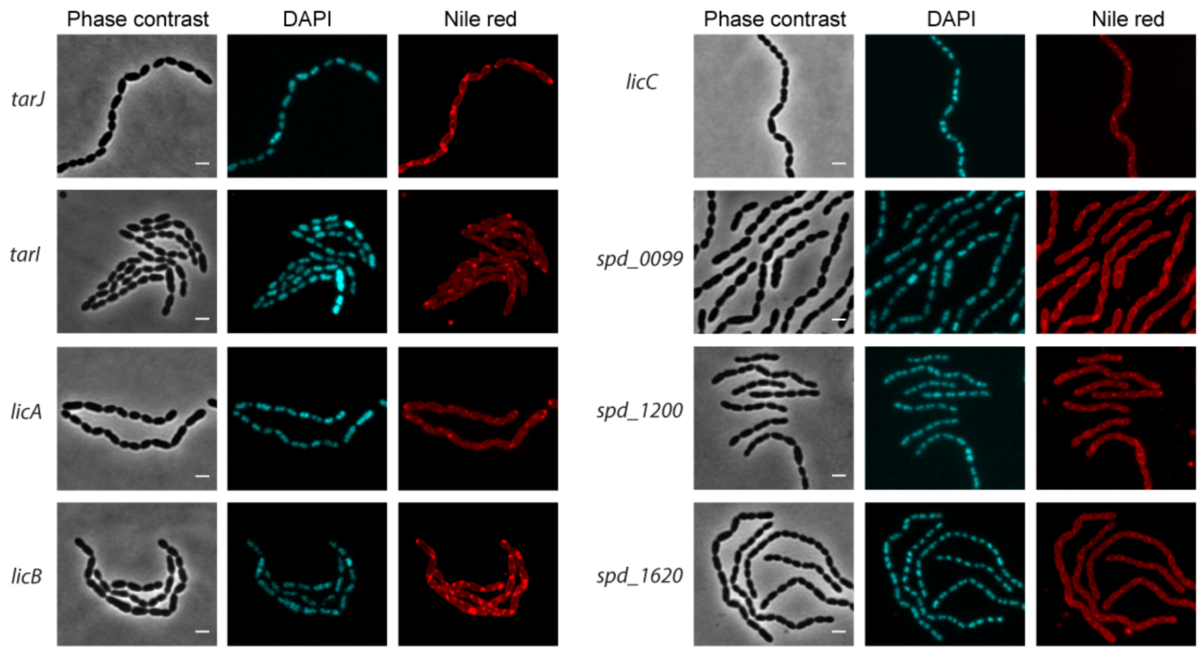

Figure S9. Microscopy of CRISPRi strains with sgRNA targeting genes involved in teichoic acid biosynthesis. Representative images of phase contrast, DAPI staining and nile red staining are shown. Scale bar = 2 μm.

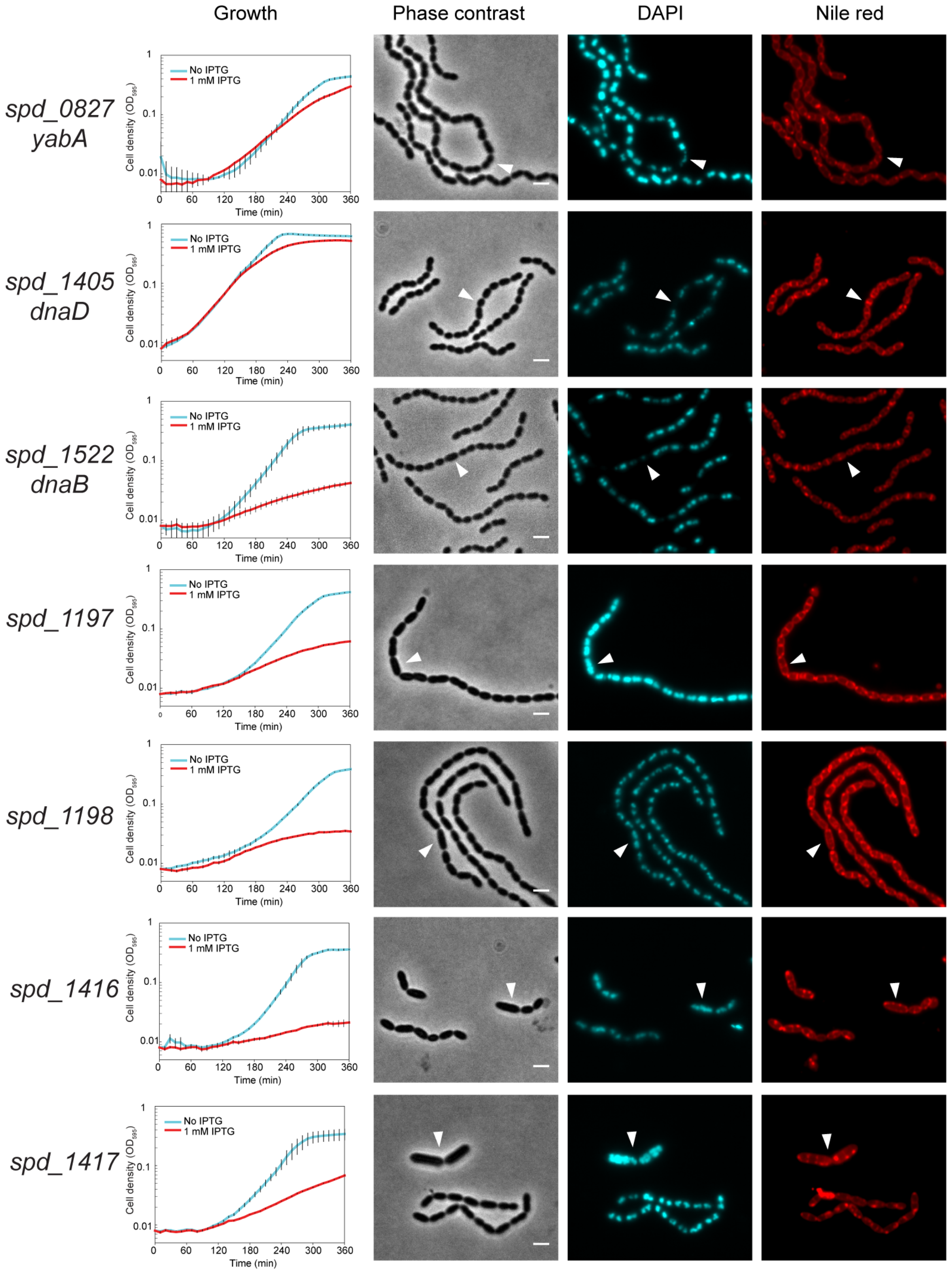

Figure S10. Growth profiles and morphological changes of CRISPRi strains with sgRNA targeting the hypothetical genes studied in this work. Growth of *S. pneumoniae* strains was performed in C+Y medium with (red lines) or without (cyan lines) 1 mM IPTG, and the averages of three replicates with SEM were used for plotting. Cells were incubated in C+Y medium with 1 mM IPTG for 2.5 hours before imaging. Representative images of phase contrast, DAPI staining and nile red staining are shown. Scale bar = 2 μm. White arrows point to cells with significant morphological changes.

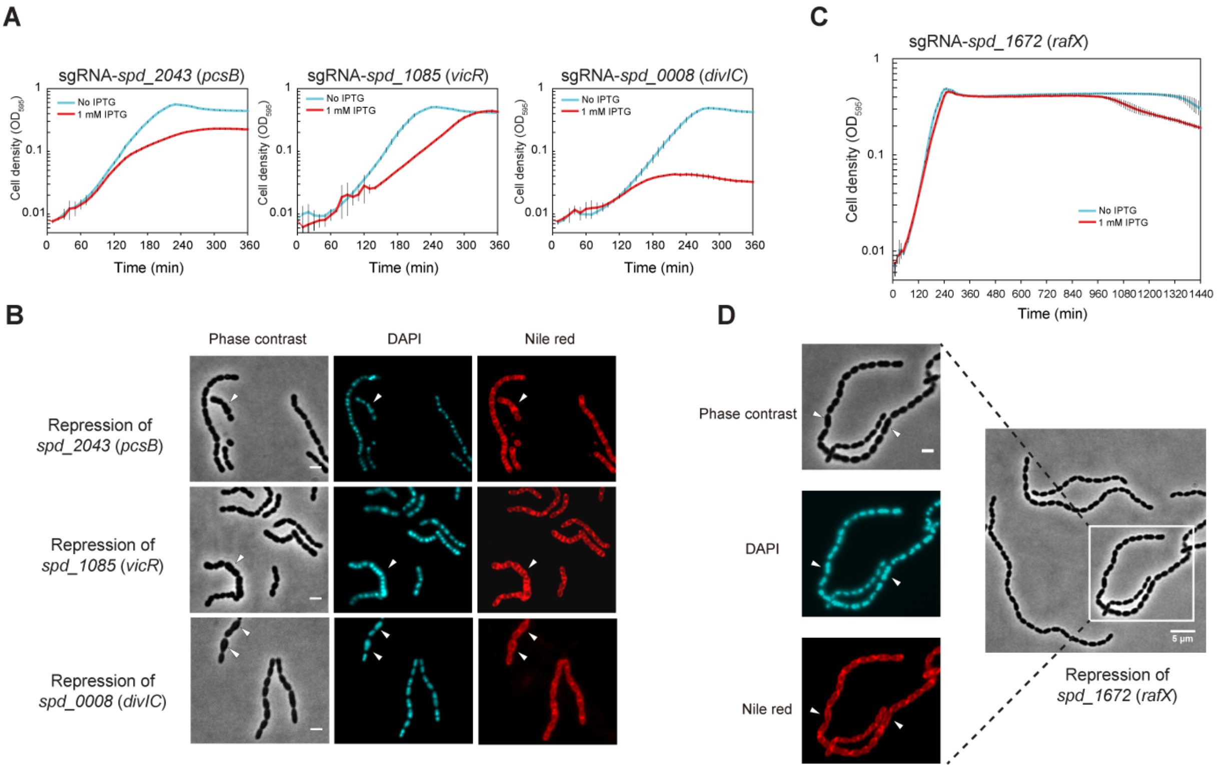

Figure S11. Functional verification of *pcsB*, *vicR, divIC and rafX* by CRISPRi.

A. Growth of CRISPRi *S. pneumoniae* strains with sgRNA targeting *spd_2043* (*pcsB*), *spd_1085 (vicR)* and *spd_0008 (divIC)* in C+Y medium with (red lines) or without (cyan lines) 1 mM IPTG. Optical density (OD_{595}) of the bacterial culture was measured every 10 minutes. Note that induction of CRISPRi targeting each of the three genes led to a significant growth defect.

B. Microscopy of CRISPRi *S. pneumoniae* strains in panel A. Representative images of phase contrast, DAPI staining and nile red staining are shown. Scale bar = 2 µm. White arrows point to typical morphological defects.

C. Repression of *spd_1672* (*rafX*) with CRISPRi leads to an increase in lysis. Growth was assayed in the same way as in panel A.

D. Repression of *spd_1672* (*rafX*) leads to longer chains and enlarged cells. Phase contrast micrograph showing a chainy phenotype is displayed on the left. Cells in the white rectangle are zoomed in, and the micrographs are displayed on the right. Phase contrast,

chromosome by DAPI staining, and cell membrane by nile red staining are shown. Scale bar = 2 μm, unless annotated.

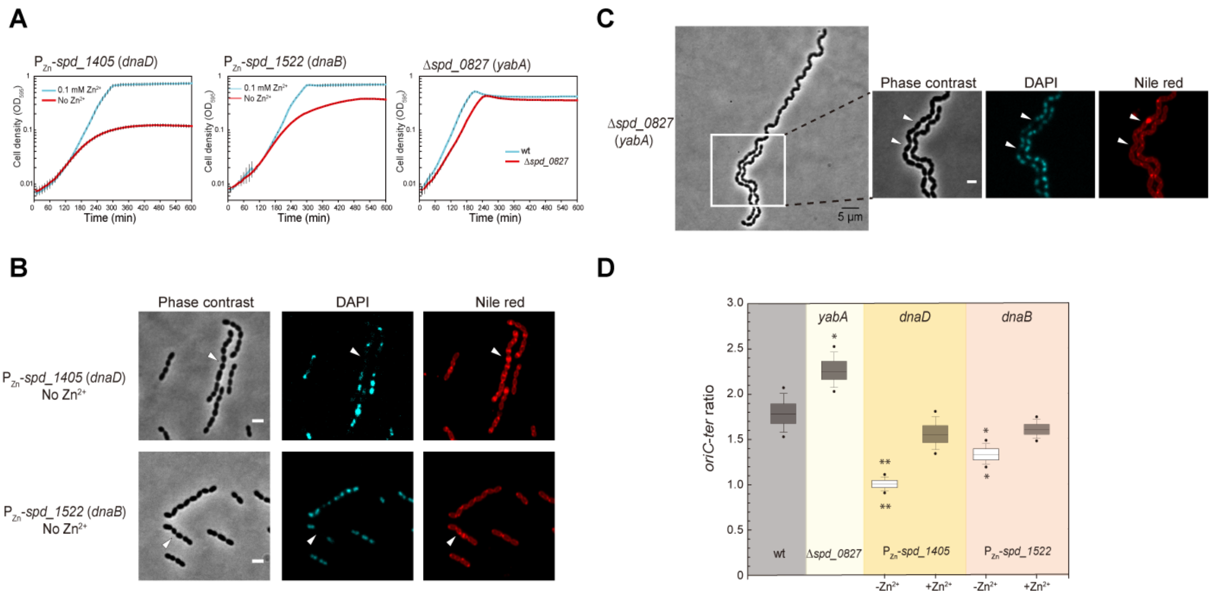

Figure S12. Annotation and characterization of *dnaD*, *dnaB*, *yabA* as important genes of chromosome biology.

A. Growth curve of Zn^{2+} inducible complementation strains of *spd_1405* (*dnaD*), *spd_1522* (*dnaB*) in medium with (cyan lines) or without (red lines) 0.1 mM Zn^{2}. For *spd_0827* (*yabA*), the knockout strain is compared with wild-type D39. Averages of three replicates with SEM were used for plotting.

B-C. Microscopy of *S. pneumoniae* strains in A. Representative images of phase contrast, DAPI staining and nile red staining are shown. Scale bar = 2 μm. White arrows point to anucleate cells. **B**. Strains with Zn^{2+} inducible complementation of *dnaD, dnaB* were incubated in C+Y medium without Zn^{2+} for 2.5 h before imaging. **C**. The knockout strain of *spd_0872* showed longer chains and appearance of anucleate cells.

D. Determination of *oriC-ter* ratio by real-time qPCR. Knockout of *spd_0827* leads to an increased *oriC-ter* ratio, whereas depletion of *spd_1405* or *spd_1522* leads to a decreased ratio. Data was processed with Monte Carlo simulations. The dots in the box plots represent the 5th and 95th percentile, and whiskers represent the 10th and 90th percentile. Asterisks on

top of the box represent significant difference from wild type strain (WT), and asterisks below the box represent significant difference from depletion strains with or without 0.1 mM Zn^{2+} (*, P<0.01; **P<0.001, p values were obtained by pairwise comparison of the 10,000 randomly simulated values of between different samples)

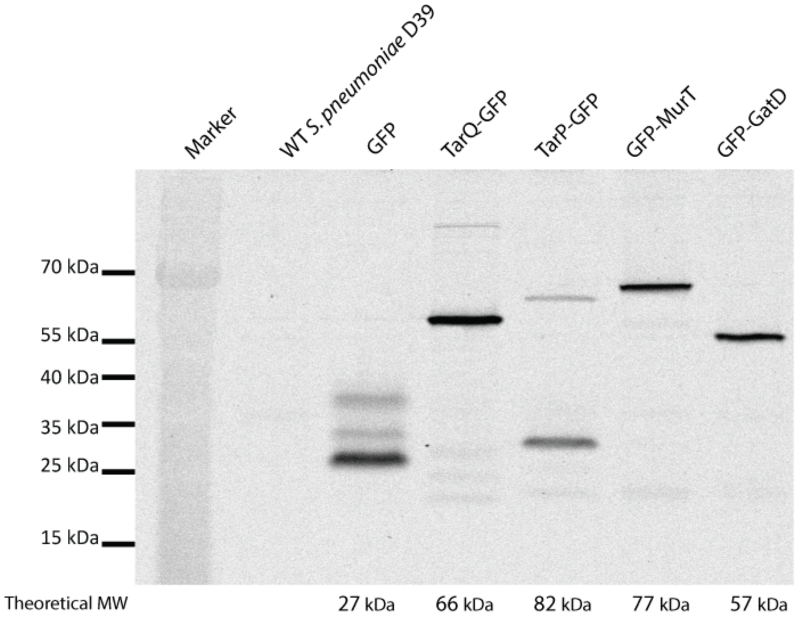

Figure S13. In-gel fluorescence scanning of TarQ-GFP, TarP-GFP, GFP-MurT and GFP-GatD. *S. pneumoniae* D39 with (GFP) or without free GFP (WT) were included as reference.

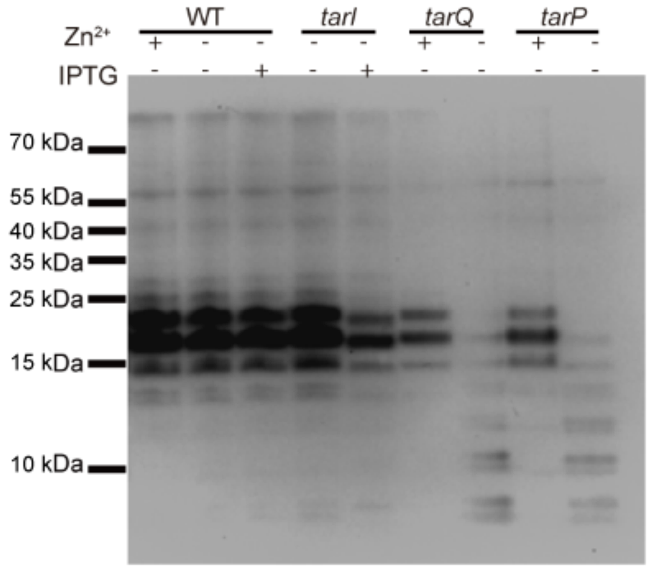

Figure S14. Western blotting to detect phosphorylcholine-containing molecules of S. pneumoniae. Related to Figure 5E. CRISPRi strain targeting *tarI,* thus repressing the

lic1 locus due to polar effects, is involved as control for the band patterning observed when depleting *tarQ* and *tarP*.

Appendix Table

Table S1. Strains and plasmids used in this study

Strains/Plasmids	Description	Reference
S. pneumoniae		
D39	Serotype 2 strain, wild-type	(Avery et al, 1944)
DCI23	D39, *ΔbgaA*::*P_{lac}-*dcas9*sp (tet^R); *ΔprsA*::F6-*lacI* (Gm^R)	This study
XL28	D39, *ΔbgaA*:: P_{lac}-*dcas9sp* (tet^R); *ΔprsA*::F6-*lacI* (Gm^R); *cil**::P3-*luc* (kan^R); *Δcep*::P3-*sgRNAluc* (spec^R)	This study
XL29	D39, *ΔbgaA*:: P_{lac}-*dcas9sp* (tet^R); *ΔprsA*::F6-*lacI*(Gm^R); *cil* *::P3-*luc* (kan^R); *Δcep*:: *MCS (spec^R)	This study
XL30	D39, *ΔbgaA*:: P_{lac}-*dcas9sp* (tet^R); *ΔprsA*::F6-*lacI* (Gm^R); *cil* *::P3-*luc* (kan^R)	This study
XL35	D39, *ΔbgaA*::P_{Zn}-*spd_1405* (tet^R)	This study
XL38	D39, *ΔbgaA*::P_{Zn}-*spd_1522* (tet^R)	This study
XL39	D39, Δ*spd_1672* (ery^R)	This study
XL44	D39, *ΔbgaA*::P_{Zn}-*spd_1416* (with C-ter fused GFP) (tet^R), Δ*spd_1416* (ery^R)	This study
XL47	D39, *ΔbgaA*::P_{Zn}-*spd_1405* (tet^R), Δ*spd_1405* (ery^R)	This study
XL48	D39, *ΔbgaA*::P_{Zn}-spd_*1522* (tet^R), Δ*spd_1522* (ery^R)	This study
CG60	D39, *ΔbgaA*::P_{Zn}-*spd_1197-gfp* (C-ter fused GFP) (tet^R)	This study
CG80	D39, *ΔbgaA*::P_{Zn}-*spd_1197-gfp* (C-ter fused GFP) (tet^R), Δ*spd_1197* (ery^R)	This study
CG144	D39, *ΔbgaA*::P_{Zn}-*spd_1198-gfp* (C-ter fused GFP) (tet^R)	This study
XL43	D39, *ΔbgaA*::P_{Zn}-*spd_1198-gfp* (C-ter fused GFP) (tet^R), Δ*spd_1198* (ery^R)	This study
XL59	D39, *ΔbgaA*::P_{Zn}-*spd_1417* (N-ter fused GFP) (tet^R), Δ*spd_1417* (ery^R)	This study
XL58	D39, *ΔbgaA*::P_{Zn}-*gfp-spd_1416* (N-ter fused GFP) (tet^R), Δ*spd_1416* (ery^R)	This study
XL100	D39, Δ*metA* (ery^R)	This study
XL101	D39, Δ*hpt* (ery^R)	This study
XL102	D39, Δ*spd_1895* (ery^R)	This study
XL103	D39, Δ*spd_0245* (ery^R)	This study
XL104	D39, Δ*spd_1520* (ery^R)	This study
XL105	D39, Δ*spd_0379* (ery^R)	This study
MK134	D39, P_{ssbB} -*luc*, *kan^R*	(Slager et al, 2014)
E. coli		
MC1061	*araD139*, *Δ(ara, leu)7697*, *ΔlacX74*, *galU-*, *galK-*, *hsr-*, *hsm+*, *strA*	(Casadaban & Cohen, 1980)
**Plasmids**		
pPEP1	*cam^R, cep'*, *spec^R*-MCS,*'cep*	(Sorg et al, 2015)
pPEPX	*cep'*, *spec^R*-MCS,*'cep*	This study
pPEP23	*cam^R, cep'*, *spec^R*-P3-*luc*,*'cep*	(Sorg et al, 2015)
pPEPX-P3-sgRNA*luc*	*cep'*, *spec^R*-P3-sgRNA*luc*,*'cep*	This study
pMK17	*amp^R*, *bgaA'*, *tet^R*, P_{Zn}-*gfp*-MCS, *'bgaA* (For N-terminal GFP fusion cloning)	This study

pCG6	*amp*^{R}, *bgaA'*, *tet*^{R}, P_{Zn}-MCS-*gfp*, *'bgaA* (For C-terminal GFP fusion cloning)	(Gallay,Veening, unpublished)
pPEPY	*cil**', *kan*^{R}, MCS, *'cil* *	(Keller,Veening, unpublished)
pJWV102	*amp*^{R}, *bgaA'*, *tet*^{R}, P_{Zn}-*gfp*, *'bgaA*	This study
pJWV102-PL-*dcas9sp*	*amp*^{R}, *bgaA'*, *tet*^{R}, P_{lac}-*dcas9sp*, *'bgaA*	This study
pMK11	*amp*^{R}, *bgaA'*, *tet*^{R}, P_{Zn}, *'bgaA*	(Kjos et al, 2016)
pJWV502	*amp*^{R}, *bgaA'*, *tet*^{R}, P_{Zn}-*gfp*, *ery*^{R}, *'bgaA*	This study
pPEPY-*lacI*-GmR	*kan*^{R}, *prsA'*, P3-*lacI*, *Gm*^{R}, *'prsA*	This study

Notes:

1. *cil: chromosome intergration locus, represents a non-coding region between *spd_0422* and *spd_0423*.
2. *MCS: multiple cloning site
3. *P_{lac}: The IPTG inducible promoter.
4. amp^R: ampicillin resistance; cam^R: chloramphenicol resistance; ery^R: erythromycin resistance; Gm^R: gentamycin resistance; kan^R: kanamycin resistance; spec^R:spectinomycin resistance; tet^R: tetracycline resistance.

Appendix References

Aranda PS, LaJoie DM, Jorcyk CL (2012) Bleach gel: a simple agarose gel for analyzing RNA quality. *Electrophoresis* **33:** 366-369

Avery OT, Macleod CM, McCarty M (1944) Studies on the Chemical Nature of the Substance Inducing Transformation of Pneumococcal Types : Induction of Transformation by a Desoxyribonucleic Acid Fraction Isolated from Pneumococcus Type Iii. *The Journal of experimental medicine* **79:** 137-158

Beilharz K, van Raaphorst R, Kjos M, Veening JW (2015) Red Fluorescent Proteins for Gene Expression and Protein Localization Studies in *Streptococcus pneumoniae* and Efficient Transformation with DNA Assembled via the Gibson Assembly Method. *Applied and environmental microbiology* **81:** 7244-7252

Casadaban MJ, Cohen SN (1980) Analysis of gene control signals by DNA fusion and cloning in *Escherichia coli*. *J Mol Biol* **138:** 179-207

de Jong A, van der Meulen S, Kuipers OP, Kok J (2015) T-REx: Transcriptome analysis webserver for RNA-seq Expression data. *BMC genomics* **16:** 663

Kjos M, Miller E, Slager J, Lake FB, Gericke O, Roberts IS, Rozen DE, Veening JW (2016) Expression of *Streptococcus pneumoniae* Bacteriocins Is Induced by Antibiotics via Regulatory Interplay with the Competence System. *PLoS pathogens* **12:** e1005422

RStudio RT. (2015) Integrated Development for R. RStudio. Inc.

Slager J, Kjos M, Attaiech L, Veening JW (2014) Antibiotic-induced replication stress triggers bacterial competence by increasing gene dosage near the origin. *Cell* **157:** 395-406

Sorg RA, Kuipers OP, Veening JW (2015) Gene Expression Platform for Synthetic Biology in the Human Pathogen *Streptococcus pneumoniae*. *ACS synthetic biology* **4:** 228-239
