# Supplementary material for: High‐throughput CRISPRi phenotyping identifies new essential genes in Streptococcus pneumoniae
Source: Mol Syst Biol. 2017 May 10;13(5):931. doi: 10.15252/msb.20167449 (PMC5448163; doi:10.15252/msb.20167449)
Supplement: Supplementary file 2 — Expanded View Figures PDF [file MSB-13-931-s002.pdf]

Expanded View Figures

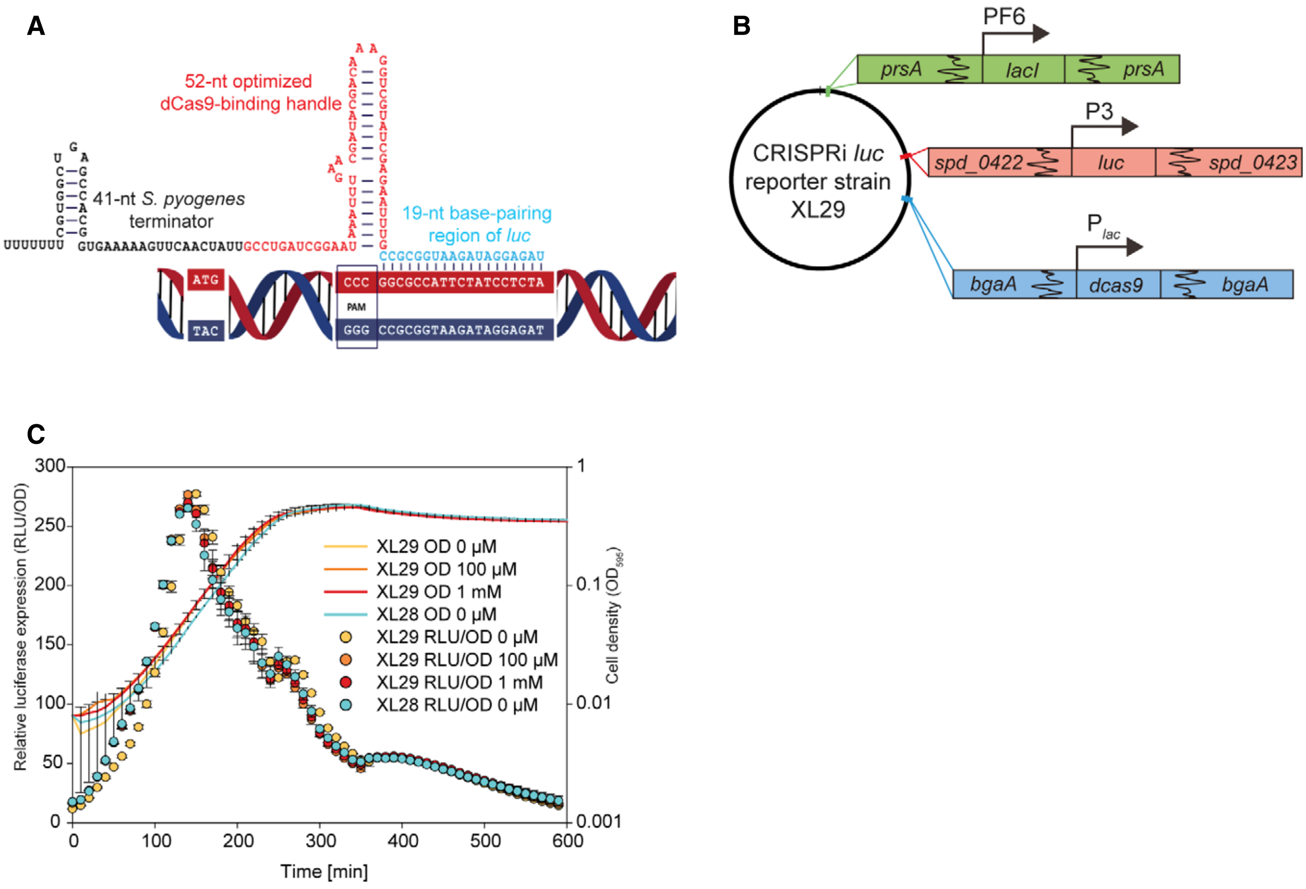

Figure EV1. Properties of the designed IPTG-inducible CRISPRi system. Related to Fig 1.

A Secondary structure of the complex of sgRNA*luc* binding to the 5′ end encoding sequence of the *luc* gene.

B Genetic map of CRISPRi *luc* reporter strain XL29. Genetic map of strain XL28 is shown in Fig 1B. Strain XL29 is genetically identical to XL28 but lacking the sgRNA*luc*.

C The CRISPRi system is tightly controlled by IPTG. Luminescence and OD_{595} were measured every 10 min, and averages of three replicates with SEM were used for plotting. Note that, in XL29, induction of dCas9 without sgRNA*luc* did not influence growth or *luc* expression. In XL28, without addition of IPTG, no repression on *luc* was observed compared with XL29.

© 2017 The Authors

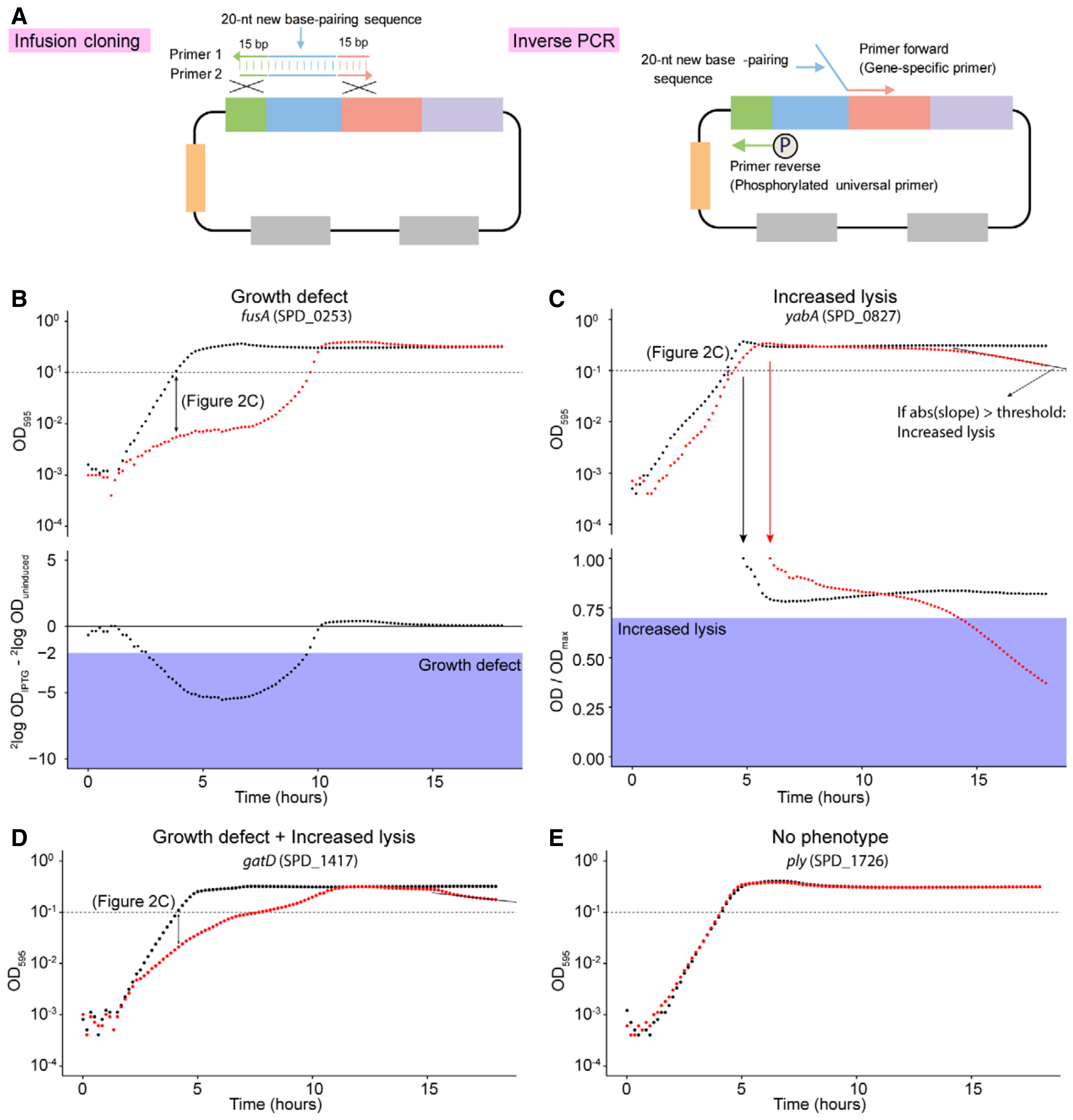

Figure EV2. Schematic of the inverse PCR cloning strategy and growth analysis of the CRISPRi library. Related to Fig 2.

A Schematic of the infusion cloning and comparison with inverse PCR. For the infusion method used for sgRNA cloning, two gene-specific primers were designed for each cloning. Primer 1 and primer 2 are complementary, and they contain 15-bp homology sequences to the adjoining region, flanking the 20-bp base-pairing region of the sgRNA encoding sequence of the vector. For inverse PCR, a 20-nt new base-pairing sequence was included in the forward gene-specific primer. The universal reverse primer was phosphorylated, to allow circularization of the vector by ligation after amplification.

B–E Definition of the growth phenotype classification utilized in Fig 2B. The data points used in Fig 2C are indicated by double-headed arrows. (B) Classification as OD-difference phenotype (growth defect), exemplified by the *fusA* knockdown dataset. Growth curves of IPTG-induced and uninduced cells are shown in the top graph. In the bottom graph, the difference in $\log_2(OD_{595})$ between IPTG-induced and uninduced cells is plotted for each time point. Datasets that contain points in the shaded area (i.e., having a > fourfold difference) are classified as having a significant growth defect. (C) Classification of the increased-lysis phenotype exemplified by the *yabA* knockdown dataset. Growth curves of IPTG-induced and uninduced cells are shown in the top graph. A best-fit straight line is created using the last 10 data points of the IPTG-treated cells (i.e., last 90 min). If the slope of this line is more negative than 0.05 h^{-1}, the strain is classified as having an increased-lysis phenotype. Additionally, datasets were normalized to the maximum OD_{595} reached, plotted in the bottom graph. When the normalized values of the IPTG-induced cells fall below 0.7 (i.e., 70% of OD_{max}), the strain is also classified as having an increased-lysis phenotype (shaded area). The black arrow points to the normalized dataset of bacterial growth in IPTG free medium, while the red arrow points to the normalized dataset of bacterial growth in medium with 1 mM IPTG. Note that while the example used (*yabA*) fulfills both criterions, fulfilling one of them is sufficient. Panels (D and E) show examples of "growth defect and increased lysis" and "No phenotype", respectively, based on the criteria demonstrated in panels (B and C).

 © 2017 The Authors

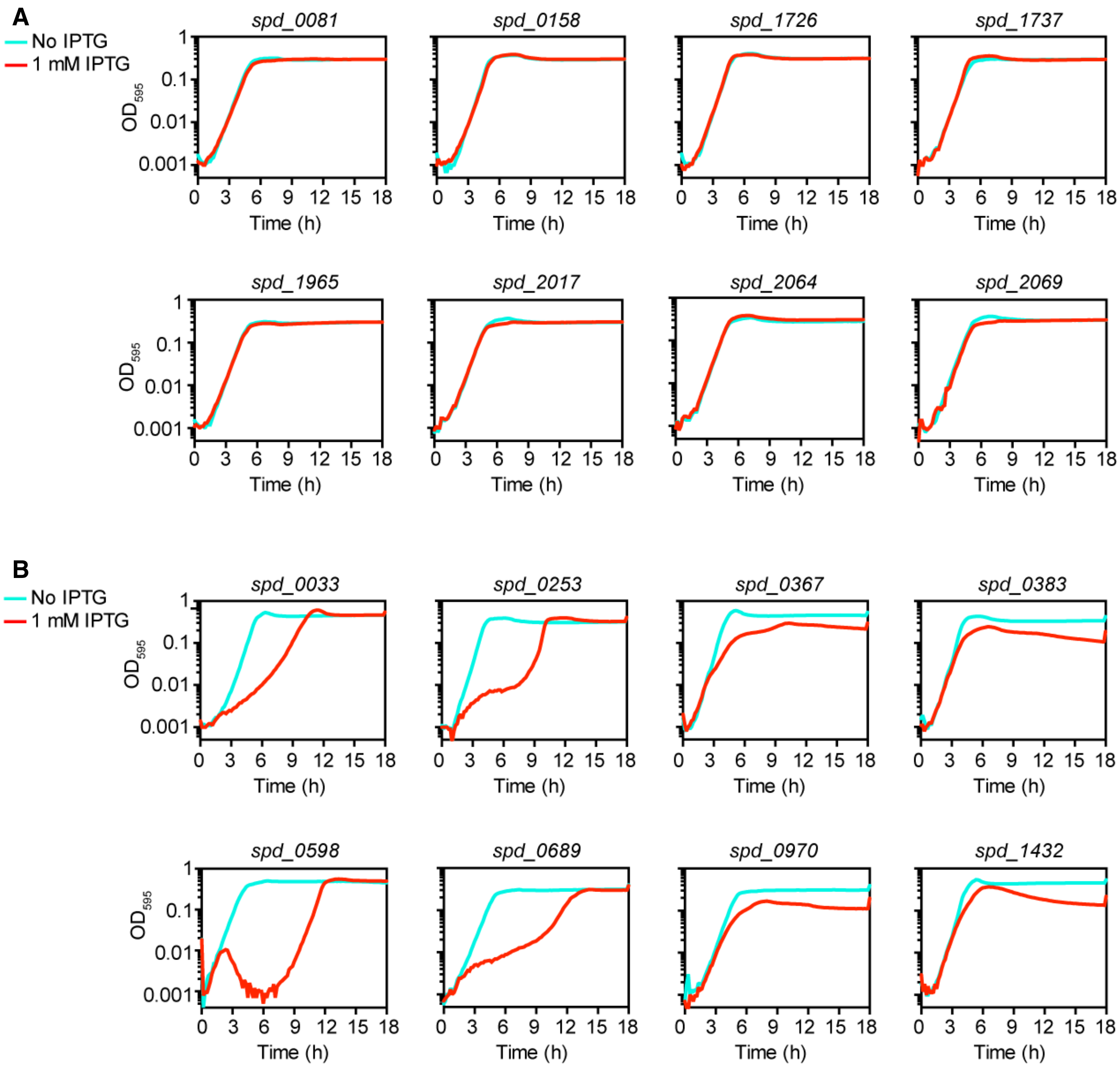

Figure EV3. Growth of CRISPRi strains targeting dispensable or essential genes.

A Growth curves of eight CRISPRi strains targeting dispensable genes. Growths of CRISPRi strains were performed in C+Y medium with (red lines) or without (cyan lines) 1 mM IPTG.

B Growth curves of eight CRISPRi strains targeting essential genes, performed similarly to panel (A).

© 2017 The Authors

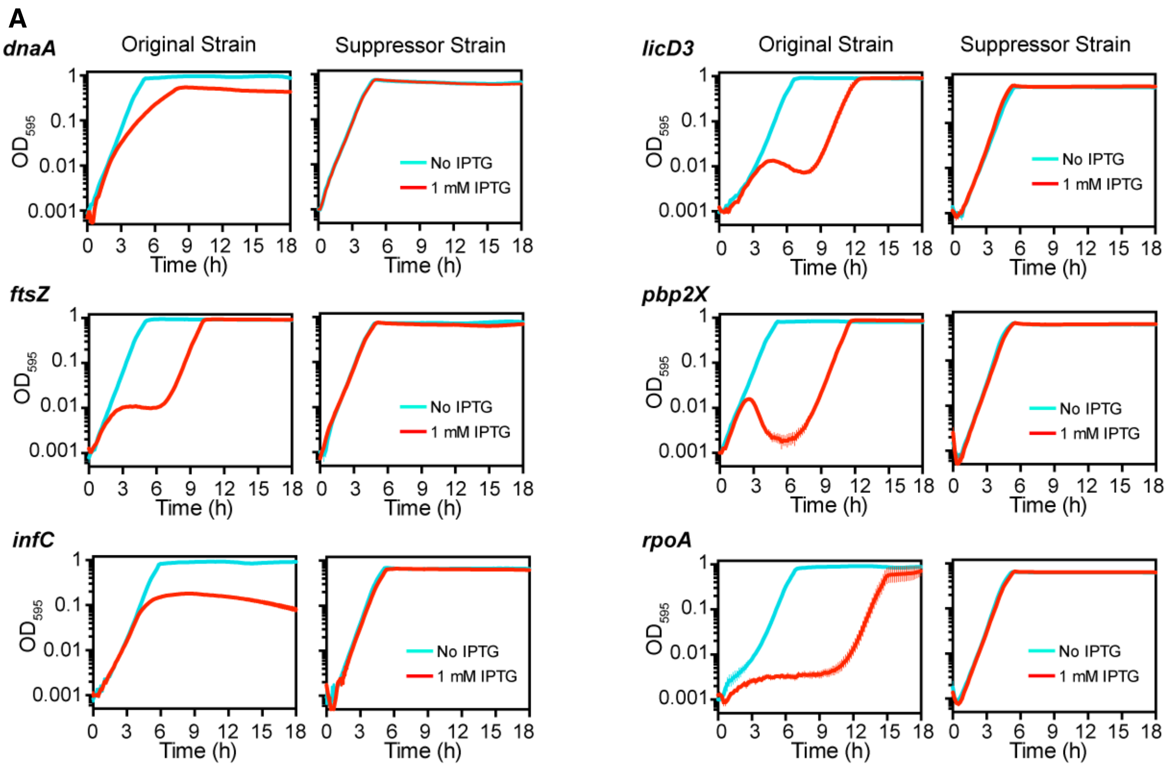

B

Target gene	sgRNA	P_{lac}-*dcas9*
dnaA	No loss-of-function mutation	S6L
ftsZ	No loss-of-function mutation	Q544*
infC	No loss-of-function mutation	Q354*
licD3	No loss-of-function mutation	Frame-shifting
pbp2X	No loss-of-function mutation	Cannot be PCR amplified
rpoA	No loss-of-function mutation	No mutation

Figure EV4. Suppression mutation of the CRISPRi system.

A Growth of original CRISPRi strains and the corresponding suppressor strains. Original strains are the strains in the CRISPRi library, which were cultured and stored in C+Y medium in the absence of IPTG. Suppressor strains were purified from the long-incubation bacterial culture of original strains in C+Y medium with 1 mM IPTG (see Appendix Methods). Strains were grown in C+Y medium with (red) or without (cyan) 1 mM IPTG. The cell density was measured every 10 min for 18 h. The values represent averages of three replicates with SEM.

B Sequencing analysis of the sgRNA and P_{lac}-*dcas9* of the suppressor strains. The sgRNA and P_{lac}-*dcas9* DNA fragment were amplified from genomic DNA of the suppressor strains. * represents stop codon.

 © 2017 The Authors

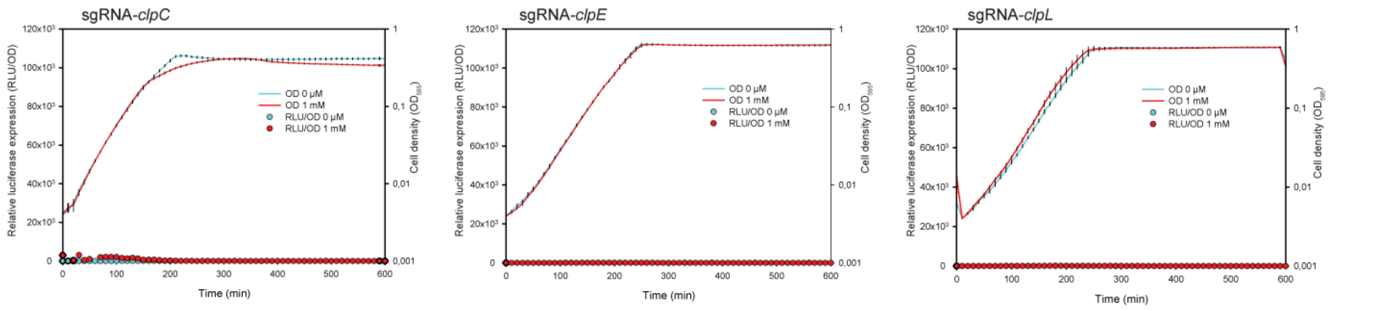

Figure EV5. CRISPRi repression of *clpC*, *clpE*, and *clpL* does not induce competence development. Related to Fig 6.
Competence development was followed using the *ssbB_luc* transcriptional fusion. The assay was performed in C+Y medium at a pH in which natural competence of the wild-type strain does not develop. 1 mM IPTG (red) or no IPTG (cyan) was added into the medium at the beginning of detection. Cell density (OD_{595}) and luciferase activity of the bacterial culture were measured every 10 min. The values represent averages of three replicates with SEM.
